# Identification of Endoplasmic Reticulum Stress-Related Biomarkers in Coronary Artery Disease

**DOI:** 10.1155/2024/4664731

**Published:** 2024-07-03

**Authors:** Yuanyuan Lin, Lin Ni, Luqun Yang, Hao Li, Zelin Chen, Yuping Gao, Kaiyi Zhu, Yanni Jia, Zhifang Wu, Sijin Li

**Affiliations:** ^1^ Department of Nuclear Medicine First Hospital of Shanxi Medical University Shanxi Medical University, Taiyuan, Shanxi 030001, China; ^2^ Shanxi Bethune Hospital Shanxi Academy of Medical Sciences Tongji Shanxi Hospital Third Hospital of Shanxi Medical University, Taiyuan 030032, China

## Abstract

Coronary artery disease (CAD) is caused by atherosclerotic lesions in the coronary vessels. Endoplasmic reticulum stress (ERS) acts in cardiovascular disease, and its role in CAD is not clear. A total of 13 differentially expressed ERS-related genes (DEERSRGs) in CAD were identified. Functional enrichment analysis demonstrated the DEERSRGs were mainly enriched in endoplasmic reticulum (ER)-related pathways. Then, eight genes (RCN2, HRC, DERL2, RNF183, CRH, TMED2, PPP1R15A, and IL1A) were authenticated as ERS-related biomarkers in CAD by least absolute shrinkage and selection operator (LASSO). The receiver operating characteristic (ROC) analysis showed that the LASSO logistic model constructed based on biomarkers had a better diagnostic effect, which was confirmed by the ANN and GSE23561 datasets. Also, ROC results showed that seven of the eight biomarkers had better diagnostic effects. The nomogram model had good predictive power, and biomarkers were mostly enriched in pathways associated with CAD. The biomarkers were significantly associated with 10 immune cells, and RCN2, DERL2, TMED2, and RNF183 were negatively correlated with most chemokines. Eight biomarkers had significant correlations with both immunoinhibitors and immunostimulators. In addition, eight biomarkers were significantly different in both CAD and control samples, CRH and HRC were upregulated in CAD. The quantitative reverse transcription-polymerase chain reaction (qRT-PCR) showed that RCN2, HRC, DERL2, CRH, and IL1A were consistent with the bioinformatics analysis. RCN2, HRC, DERL2, RNF183, CRH, TMED2, PPP1R15A, and IL1A were identified as biomarkers of CAD. Functional enrichment analysis and immunoassays for biomarkers provide new ideas for the treatment of CAD.

## 1. Introduction

Coronary artery disease (CAD) is characterized by myocardial ischemia, hypoxia, or necrosis, resulting from coronary atherosclerosis and vascular stenosis or obstruction [1, 2]. Clinically, current diagnosis of CAD primarily relies on medical history, blood tests, electrocardiogram (ECG), echocardiography, coronary angiography, CT angiography, and cardiac catheterization [3]. However, these methods have limitations in terms of accuracy, invasiveness, and cost-effectiveness. Moreover, due to the side effects of drug treatments, the restenosis rate following interventional procedures, and the risks associated with surgical interventions, highlighted the necessity of discovering more effective and safer diagnostic and treatments [3, 4]. It is crucial for enhancing the prognosis and quality of life for patients with CAD.

CAD is a heterogeneous disease that exhibits varied manifestations in individuals, influenced by multiple factors, including genetics, environment, and lifestyle. The underlying pathophysiology of CAD involves various cellular and molecular interactions, such as inflammation, oxidative stress, lipid metabolism, endothelial dysfunction, and vascular remodeling [5]. In the early stages, detecting CAD can be challenging without specific screening programs. While there are some biomarkers associated with CAD, such as high-sensitivity C-reactive protein (hsCRP), high-sensitive cardiac troponin, and lipid levels, these biomarkers are not specific to CAD and may not be sufficient for diagnosis on their own [6]. Early detection is crucial for timely intervention and prevention of complications [7]. New biomarkers that can detect CAD in its early stages would be highly beneficial [8]. The use of biomarkers in CAD diagnosis can lead to more personalized treatment approaches, targeting the underlying mechanisms of the disease in individual patients [9].

Endoplasmic reticulum stress (ERS) is a cellular stress response that involves the protein folding system in the endoplasmic reticulum (ER). ERS is triggered when cells are subjected to a variety of subcellular stresses, such as ischemia-reperfusion injury, drug effects, and oxidative stress. ERS promotes cellular adaptation to stressful environments by activating different signaling pathways and maintains protein homeostasis, but it can also cause cell death in case of stress overload [10]. ERS plays a crucial role in the progression of CAD by promoting inflammation and additional related mechanisms. Atherosclerosis is the main pathological basis of CAD. ER stress has been implicated in the development of atherosclerosis [11]. ER stress in endothelial cells, smooth muscle cells, and macrophages within the arterial wall can promote inflammation, oxidative stress, and cell death, all of which contribute to plaque formation and progression [11]. ER stress in hepatocytes can disrupt lipid metabolism, leading to dyslipidaemia, which is a major risk factor for CAD [12]. ER stress can increase the synthesis and secretion of lipids and lipoproteins, contributing to the formation of atherosclerotic plaques. ER stress in vascular smooth muscle cells can promote vascular remodeling and vasoconstriction, which can contribute to the development of CAD. ER stress in macrophages within atherosclerotic plaques can promote plaque instability and rupture, leading to acute cardiovascular events [13]. ER stress in platelets and endothelial cells can promote thrombosis, which can further obstruct coronary arteries and lead to acute cardiovascular events [14]. In summary, ER stress plays a significant role in the pathogenesis of CAD by contributing to atherosclerosis, dyslipidaemia, insulin resistance, vascular dysfunction, plaque instability, and thrombosis. Targeting ER stress pathways may offer new therapeutic approaches for the prevention and treatment of CAD.

This study is aimed at exploring the role of ER stress in the initiation, progression, or prognosis of CAD. Using the Gene Expression Omnibus (GEO) database, bioinformatics analysis revealed correlations between the genes and CAD. The study identified ERS-related genes (ERSRGs) that were highly expressed in CAD and explored their association with immune cells through immune infiltration analysis. These findings will provide a basis to further elucidating the molecular mechanism of ER stress in the pathogenesis of CAD.

## 2. Materials and Methods

### 2.1. Data Source

Two independent cohorts of CAD (GSE113079 and GSE23561) were obtained from the GEO database (https://www.ncbi.nlm.nih.gov/geo/). The GSE113079 dataset contains microarray data of peripheral blood mononuclear cell (PBMC) samples from 93 CAD samples and 48 control samples and was used as a training set [15]. The GSE23561 dataset as a validation set contains 35 samples; we have chosen six CAD samples and nine control peripheral blood samples for further analysis [16]. A total of 439 ERSRGs were acquired from the GeneCards database (https://www.genecards.org) by setting the criterion as relevance scores ≥ 10 [17].

### 2.2. Identification and Functional Enrichment Analyses of Differentially Expressed ERSRGs (DEERSRGs)

The differentially expressed genes (DEGs) between two groups (CAD vs control) in the GSE113079 dataset were screened by limma package (|log_2_FC| >1, adj.*p* < 0.05) [18]. The volcano maps were plotted by the ggplot2 (version 3.3.5) R package to present the screening results, and the pheatmap (version 1.0.12) R package was used to plot heatmaps of DEGs. DEERSRGs were obtained by intersecting the DEGs between two groups with 439 ERSRGs by the ggvenn R package. Analyses of Gene Ontology (GO) and Kyoto Encyclopedia of Genes and Genomes (KEGG) enrichment were performed on the DEERSRGs by the R language clusterProfiler (*p* < 0.05) [19].

### 2.3. Construction and Validation of Least Absolute Shrinkage and Selection Operator (LASSO) Logistic Models

In the GSE113079 dataset, DEERSRG was analyzed using the glmnet package and biomarkers were obtained using the LASSO algorithm [20]. The LASSO logistic model was constructed based on biomarkers, and artificial neural network (ANN) was constructed using the neuralnet R package to validate the model. In the validation set, receiver operating characteristic (ROC) curves of the model and biomarkers were plotted to validate the diagnostic capabilities of the models and biomarkers.

### 2.4. Construction of Nomogram

In the GSE113079 dataset, a nomogram of biomarkers was constructed using the R language rms package, and calibration curve was used to assess the reliability of the nomogram model [21]. To assess the predictive power of nomogram models, clinical impact curves (CICs) and decision curve analysis (DCA) were used.

### 2.5. Gene Set Enrichment Analysis (GSEA)

In the GSE113079 dataset, GSEA was conducted on the biomarkers based on two gene sets (c2.cp.kegg.v7.4.symbols.gmt, c5.go.bp.v7.4.symbols.gmt) using GSEA function of the clusterProfiler R package [22].

### 2.6. Analysis of Immune Infiltration and Immune Microenvironment

The immunedeconv package was used to perform cell-type identification by estimating relative subsets of RNA transcript (CIBERSORT) analysis to assess the extent of immune cell infiltration in samples from the GSE113079 dataset [23]. The Wilcoxon test was then used to obtain the differential immune cells in two groups (CAD and control). Then, Pearson's analysis calculated the relationship between immune cells, chemokines, immunoinhibitors, immunostimulators, and biomarkers (|cor| > 0.3, *p* < 0.05).

### 2.7. Blood Sample Collection

Coronary angiography was performed in all patients with chest pain, and CAD was ruled out in those patients whose results showed no stenosis of coronary or a stenosis of less than 50%; these individuals served as normal samples. Patients with greater than or equal to 50% coronary artery stenosis served as the CAD group. This study was approved by the Ethics Committee of Shanxi Bethune Hospital, and the ethics approval number was YXLL-2023-238. Informed consent was obtained from all individual participants included in the study. All experiments were performed in accordance with relevant guidelines and regulations.

### 2.8. Validation of Biomarker Expression

The expression of biomarkers was verified by quantitative reverse transcription-polymerase chain reaction (qRT-PCR). The blood samples from 10 CAD samples and 10 normal samples were processed to isolate PBMCs using Ficoll solution. Total RNA was extracted from PBMCs with the TRIzol method. SureScript-First-strand-cDNA-synthesis-kit (Servicebio, China) was used to perform the reverse transcription reactions, followed by qRT-PCR using Universal Blue SYBR Green qPCR Master Mix. The qRT-PCR thermocycling protocol was initial denaturation at 95°C for 60 s, denaturation at 95°C for 20 s, annealing at 55°C for 20 s, and amplification for 40 cycles. The housekeeping gene was GAPDH. The expression levels of the genes were calculated by the 2^-△△CT^ method and normalized with GAPDH.  Table 1 shows the primer sequences.

## 3. Results

### 3.1. DEERSRGs Were Enriched in Pathways Associated With the ER

**Figure 1:**
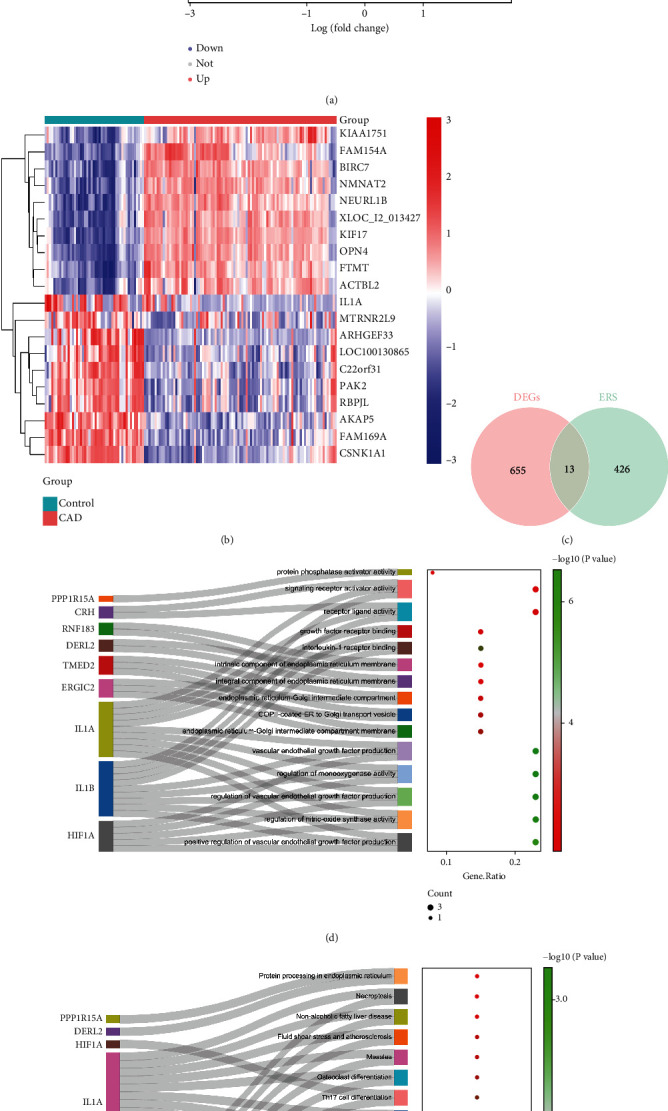
Identification of DEERSRGs and functional enrichment analysis. (a) Volcano plot of DEGs between CAD and controls. The horizontal axis is logFC, and the vertical axis is -log10 (adj. p. al). Downregulated genes are shown in blue, upregulated genes in red, and genes with insignificant differences in gray. (b) Heatmap for the top 20 DEGs between CAD and control samples. Red indicates high expression, and blue indicates low expression. (c) Venn diagram of differentially expressed ER stress genes. (d) GO enrichment results. Sankey plots of GO entries and corresponding genes are shown on the left, and bubble plots of enrichment results are shown on the right. (e) KEGG enrichment results. Sankey plots of KEGG entries and corresponding genes are shown on the left, and bubble plots of enrichment results are shown on the right.

Six hundred sixty-eight DEGs (277 downregulated and 391 upregulated) were identified between two groups (CAD vs control) in the GSE113079 dataset (Figures 1(a) and 1(b)). Six hundred sixty-eight DEGs and 439 ERSRGs were intersected to obtain 13 DEERSRGs (Figure 1(c)). The GO analysis displayed that 13 DEERSRGs mainly participated in ER lumen, response to ERS, and vascular endothelial growth factor (VEGF) production (Figure 1(d)). KEGG enrichment results demonstrated that DEERSRGs were mostly enriched in the necroptosis, cytokine-cytokine receptor interaction pathways, and protein processing in ER (Figure 1(e)).

### 3.2. Identification of Biomarkers

**Figure 2:**
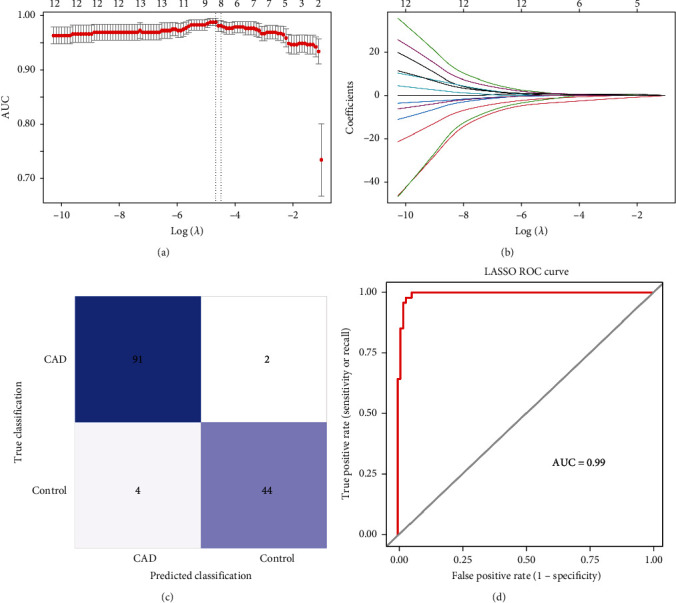
Identification of biomarkers. (a) The horizontal axis is the log lambda value, and the vertical axis is the regression coefficient. Different genes in different colors show the change of regression coefficient with the change of log lambda. (b) The horizontal axis is the log lambda value, and the vertical axis is the partial likelihood deviation value. The red dots are the variation of the partial likelihood deviation as the log lambda value is changed. The left dashed line is the position of lambda min, and the right dashed line is the position of lambda lse. (c, d) LASSO logistic model assessment. From left to right: confusion matrix heat map and ROC curve. The first grid on the upper left of the confusion matrix heat map is true positive result, the lower right is true negative result, and the upper right and lower left are false positive and false negative results. AUC, area under the curve.

**Figure 3:**
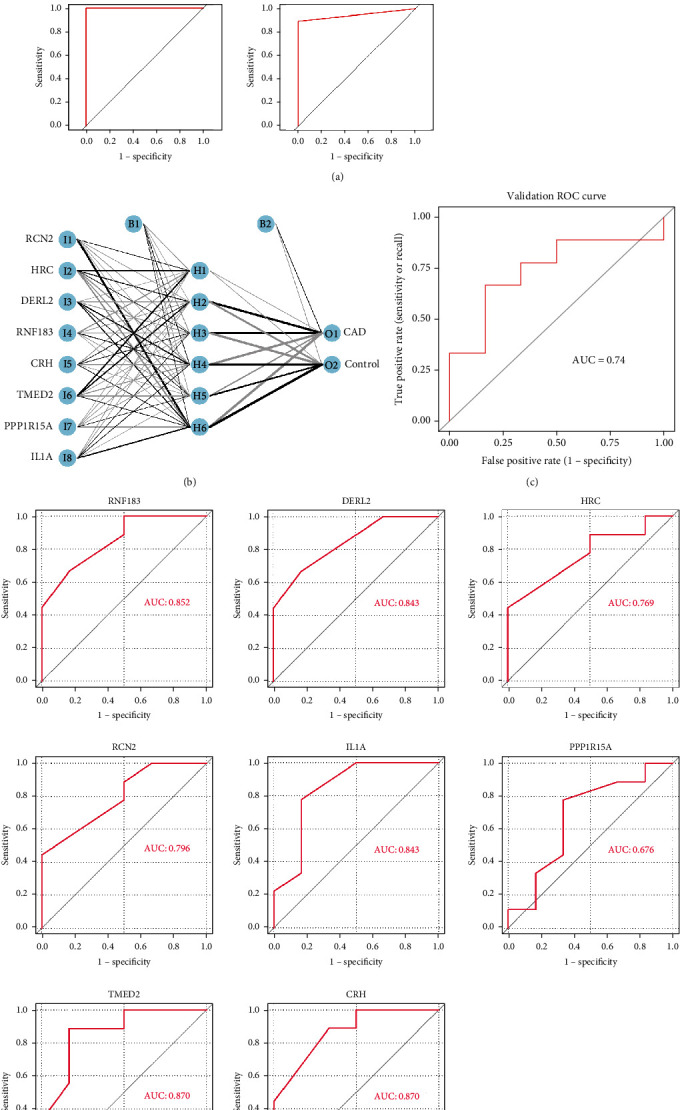
Construction of a diagnostic model using the biomarkers. (a) Validation of the diagnostic model by plotting ROC curves at one to 5 folds. (b) Artificial neural network. (c) ROC curves in the validation set. (d) ROC curves of biomarkers.

In the GSE113079 dataset, eight biomarkers (RCN2, histidine-rich calcium binding protein (HRC), DERL2, RNF183, CRH, TMED2, PPP1R15A, and IL1A) were obtained by LASSO regression analysis screening based on 13 DEERSRGs (Figures 2(a) and 2(b)). The LASSO logistic model was built based on eight biomarkers, and results of the confusion matrix heat map showed that 91 out of 93 CAD samples were correctly categorized and 44 out of 48 control samples were correctly categorized (Figure 2(c)). The area under curve (AUC) value of the LASSO logistic model was 0.99, which indicated the good predictive power of the model (Figure 2(d)). The ANN showed that the AUC values of the ROC curves were all above 0.8, indicating that the overall results of the model were good (Figures 3(a) and 3(b)). In the validation set, the AUC of the ROC curve of the model was 0.74, and the AUC values of seven of the eight biomarkers were greater than 0.7, indicating that both the model and the biomarkers had good diagnostic ability (Figures 3(c) and 3(d)).

### 3.3. Nomogram of Biomarkers

**Figure 4:**
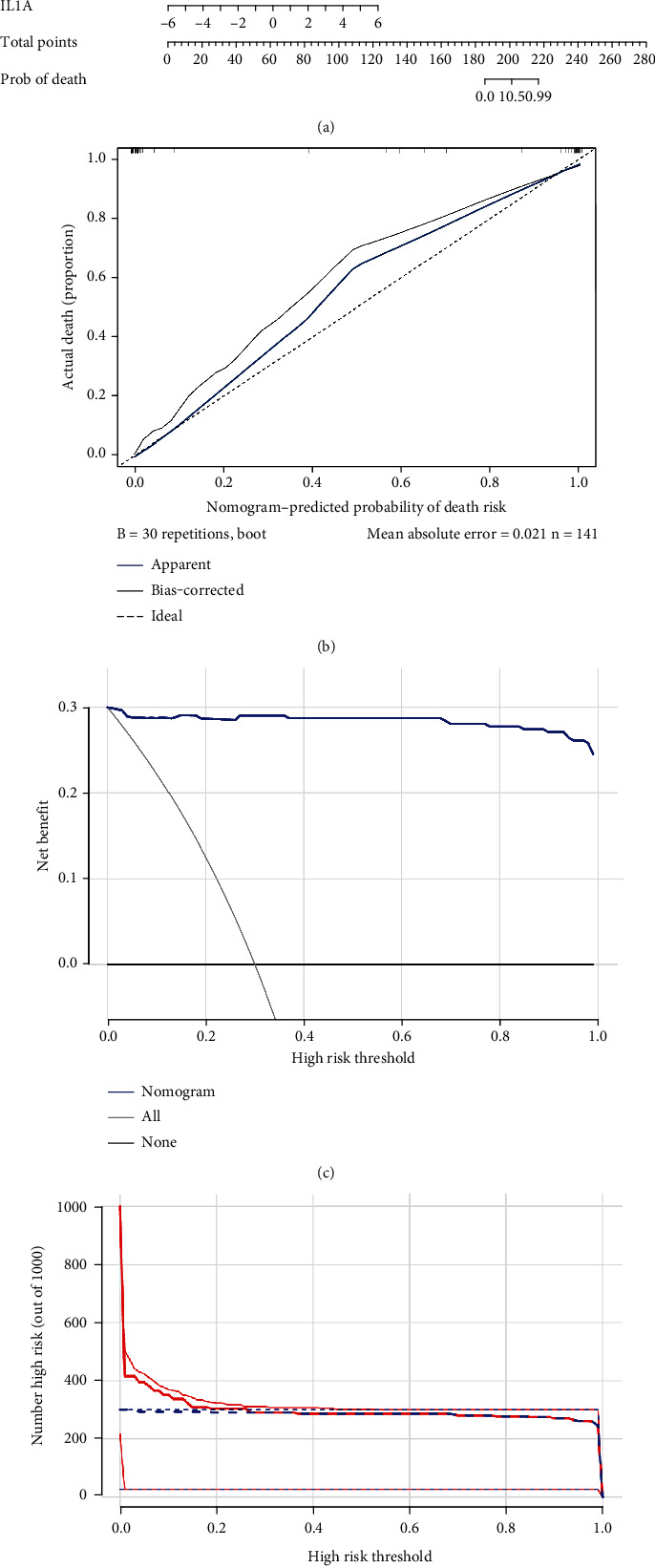
Nomogram construction and the diagnostic value evaluation. (a) The visible nomogram for diagnosing CAD. (b) The calibration plot for internal validation of the nomogram. (c) The DCA curves of the nomogram. (d) Clinical impact curves of the nomogram model.

The nomogram was constructed based on eight biomarkers (Figure 4(a)). The calibration curve revealed that nomogram was able to make accurate predictions (Figure 4(b)). In addition, the DCA curve showed that the nomogram prediction results (blue line) were significantly higher than the assumed results (gray line), indicating the good predictive performance of the model (Figure 4(c)). The CIC demonstrated that the probability threshold is after 0.2, and the judgment was highly consistent with the actual results (Figure 4(d)).

### 3.4. GSEA of Biomarkers

The GSEA results for each biomarker are described in Figures S1 and S2. In GO entries, these biomarkers were mainly enriched in DNA repair, cell cycle phase transaction, muscle contraction, and morphogenesis involved in neuronal differentiation, and other pathways are the common KEGG enriched by these biomarkers. In the KEGG enrichment results, biomarkers were enriched for the ECM receptor interaction, chemokine, and T cell receptor signaling pathway.

### 3.5. Immune-Related Analysis of Biomarkers

**Figure 5:**
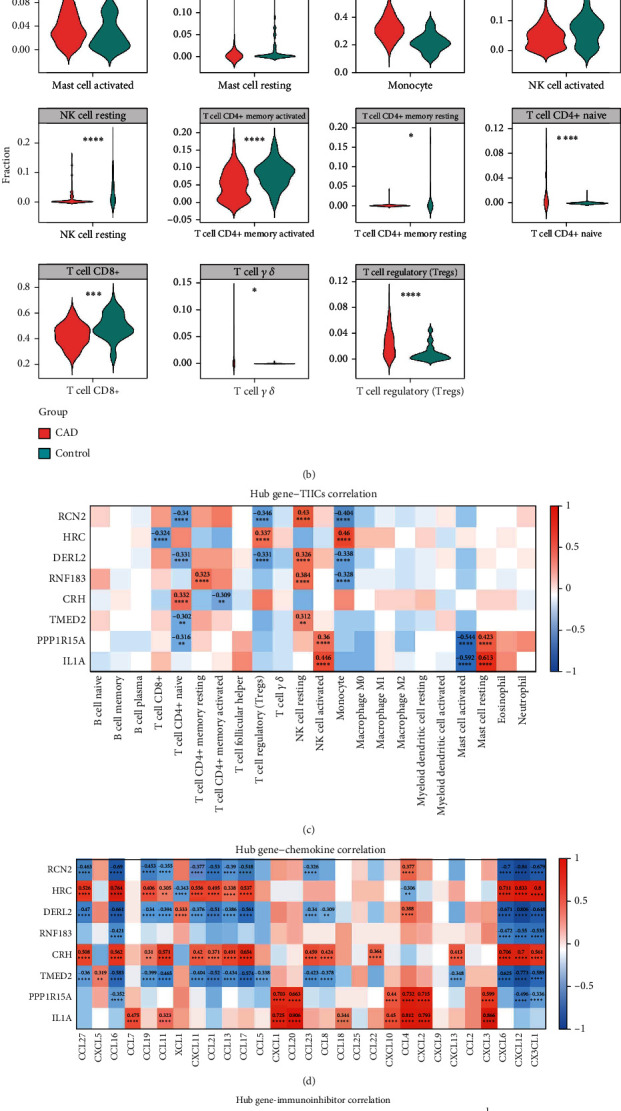
Immune-related analysis of eight DEGs. (a) CIBERSORT Stack Bar Chart. The vertical axis is a relative percentage, each column is 1 sample, and different colors distinguish 22 kinds of immune cells. (b) Differential immune cell between CAD and control sample. (c) Correlation between biomarkers and 22 kinds of immune cells. The vertical axis shows eight biomarkers, and the horizontal axis shows 22 immune cells. (d) Correlation between biomarkers and chemokines. Chemokines on the horizontal axis and biomarkers on the vertical axis. (e) The relationship between biomarker and immunosuppressants. Immunosuppressants on the horizontal axis and biomarkers on the vertical axis. (f) Correlation between biomarkers and immune activators. Immunosuppressants on the horizontal axis and biomarkers on the vertical axis. In (c–f), blue represents negative correlation, red represents positive correlation, and the number in the grid is the correlation coefficient cor.n|cor|. The larger the value, the higher the correlation. ^∗^*p* < 0.05; ^∗∗^*p* < 0.01; ^∗∗∗^*p* < 0.001; ^∗∗∗∗^*p* < 0.0001.

The role of immune cells in CAD may provide potential targets for developing novel therapeutic approaches. By studying the infiltration of immune cells, we can identify differential immune cells and explore molecular mechanisms that may influence CAD development, thereby providing a basis for designing new treatment strategies. In this study, the infiltration of 22 immune cells was detected in CAD and control samples from the GSE113079 dataset (Figure 5(a)). Additionally, the resting memory T cell CD4+, activated mast cell, naive T cell CD4+, resting mast cell, T cell CD8+, monocyte, activated memory T cell CD4+, resting NK cell, gamma delta T cell, activated NK cell, and regulatory T cell (Tregs) were found significantly different in the two groups (CAD vs control) (*p* < 0.05) (Figure 5(b)). Next, correlation analysis showed that biomarkers were significantly associated with 10 immune cells (Figure 5(c)). Among them, IL1A was significantly positively correlated with resting mast cell and with activated and negative mast cell, and RCNA2, DERL2, RNF183, and TMED2 showed a significant positive correlation with resting NK cells (Figure 5(c)).

RCN2, DERL2, TMED2, and RNF183 were negatively correlated with most chemokines (C-C chemokine ligand 2 (CCL2), CCL16, CCL19, CXCL11, CCL21, CCL13, CCL5, CCL23, CXCL6, CXCL12, CX3CL1, and CCL11), and HRC, CRH, PPP1R15A, and IL1A were positively correlated with most chemokines (Figure 5(d)). IL1A was significantly positively correlated with CCL20. RCN2 was significantly negatively correlated with CXCL12. RCN2, DERL2, RNF183, TMED2, and PPP1R15A were significantly positively correlated with immunoinhibitors (TGFBR1, CD160, and IL10RB). HRC and CRH were significantly negatively correlated with TGFBR1, CD160, and IL10RB (Figure 5(e)). RCN2 and DERL2 were significantly positively correlated with immunostimulator (CD48, TNFSF9, ICOS, CD28, and CD80), and HRC and CRH were significantly negatively correlated with CD48, TNFSF9, ICOS, CD28, and CD80 (Figure 5(f)).

### 3.6. Expression of Biomarkers in CAD and Control Samples

**Figure 6:**
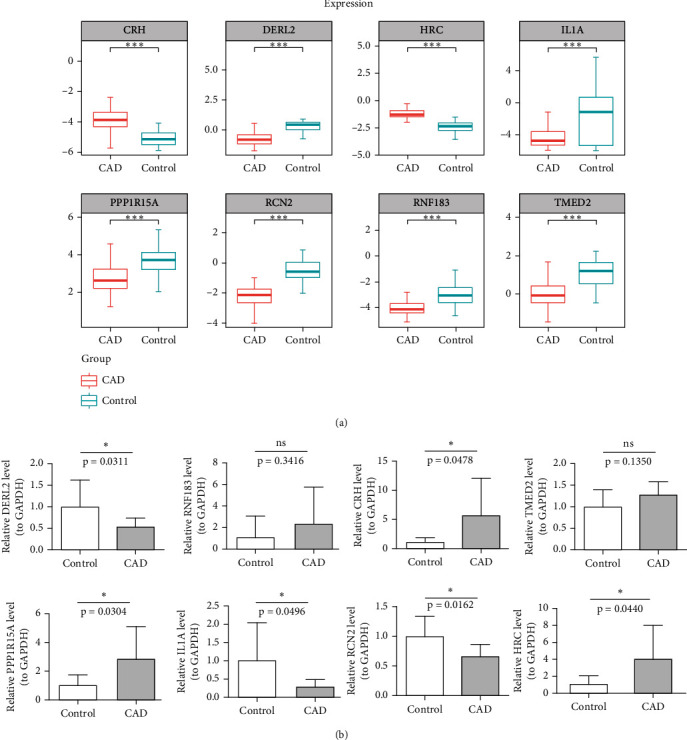
The expression levels of biomarkers in the CAD and control groups. (a) Training set; (b) qRT-PCR. ns, not significant; ^∗^*p* < 0.05; ^∗∗∗^*p* < 0.001.

In the GSE113079 dataset, CRH and HRC were upregulated in CAD and all other biomarkers were downregulated in CAD expression compared to the control group (Figure 6(a)). Moreover, we collected peripheral blood from 10 CAD patients and 10 normal individuals, and the expression levels of biomarkers were validated by qRT-PCR. The results demonstrated that the expression levels of three genes (DERL2, RCN2, and IL1A, *p* < 0.05) were decreased in the peripheral blood of CAD patients, the expression levels of three genes (CRH, PPP1R15A, and HRC, *p* < 0.05) were increased, and the expression levels of two genes did not change significantly (RNF183 and TMED2, *p* > 0.05) (Figure 6(b)).

## 4. Discussion

CAD is a condition caused by atherosclerosis in the coronary arteries, leading to narrowed blood vessels and insufficient oxygen supply to the heart muscle, resulting in myocardial ischemia and hypoxia [24]. ERS is a protective response by activating folded protein steady-state response to restore proteins [10]. Previous studies have shown that ERS plays a crucial role in cardiovascular disease, but its specific machine in CAD and whether it can be used as a biomarker for CAD are not known. Therefore, in this study, it found eight differential ERS genes (RCN2, HRC, DERL2, RNF183, CRH, TMED2, PPP1R15A, and IL1A) related to CAD genes by bioinformatics methods, which can be used as potential biomarkers of CAD. Some of these genes have been linked to atherosclerosis and myocardial infarction, offering a robust theoretical foundation for clinical research and treatment. Previous research has indicated that RCN2 is a candidate gene for atherosclerosis, contributing to vascular remodeling in hypertension [25–27]. Singh et al. have also suggested that variations in the HRC gene may be associated with the risk of myocardial infarction [28]. Additionally, the upregulation of vascular cell adhesion molecule-1 by CRH has been shown to promote atherosclerosis progression in LDL receptor-deficient mice [29]. It also laid a foundation to further explore the role of ERS in CAD mechanism and provided a new idea for diagnosis and therapy of CAD.

This study identifies 13 DEERSRGs associated with ERS and CAD. These genes are involved in crucial processes such as ER components, protein processing pathways, and vascular endothelial function regulation, providing theoretical evidence for the potential mechanisms of CAD. Specifically, in GO enrichment analysis, ERS directly related to functions of endostatin-related terms, including ER-Golgi apparatus intermediate ependyma, COPII-coated ER to Golgi matrix transport vesicles, ER-Golgi apparatus intermediate chambers, ER components, and ER protein processing pathways. Meanwhile, ERS is also enriched in the regulation and production of VEGF. In summary, VEGF and endostatin are two important factors that affect vascular endothelial function, which is consistent with previous findings [30]; therefore, their dynamic regulation plays an important role in the development of CAD. In addition, KEGG results show that necrotizing apoptosis is also closely related to CAD, and the number of vascular endothelial cell apoptosis will significantly increase in patients with early CAD [31]. At the same time, it is also enriched in the ER protein processing pathway which is related to the ERS response. In a word, the enrichment results indicated that these 13 genes were associated with ERS and CAD.

Results of the LASSO logistic model constructed for 13 genes showed that the coefficients of eight genes (RCN2, HRC, DERL2, RNF183, CRH, TMED2, PPP1R15A, and IL1A) were not penalized to zero. Therefore, eight biomarkers were identified from the 13 DEERSRGs by logistic regression. The diagnostic capabilities of these eight biomarkers were evaluated using a confusion matrix heat map and ROC curve analysis, which demonstrated good performance. Furthermore, the model was successfully validated using an external validation dataset. Among these eight genes, RCN2 was highly expressed in endothelial and atherosclerotic plaques in humans and mice [27]. Therefore, compared with healthy samples, serum or plasma RCN2 levels in atherosclerotic patients and mice prone to atherosclerosis are increased. Considering that atherosclerosis is the main pathological basis of CAD, circulating RCN2 may become a new biomarker for identifying individuals with CAD [27]. Meanwhile, further evidence supports that the upregulation of RCN2 in atherosclerosis [27], as well as the ability of IL-1A to mitigate the progression of atherosclerosis [32], can both serve as potential biomarkers for CAD. As a regulator of cardiovascular, gastrointestinal, reproductive, and immune systems, CRH plays a variety of biological effects in the cardiovascular system, such as vasodilation, positive inotropic and inotropic effects, and cardiac protection against ischemia-reperfusion injury, so it may be a potential therapeutic target for CAD, congestive heart failure, and hypertension [33]. RNF183 is mainly localized to the ER, Golgi apparatus, and lysosomes [34]. The transmembrane E3 ligase RNF183 mediates ER stress-induced apoptosis by degrading Bcl-xL [35]. As a sarcoplasmic reticulum protein, HRC mainly regulates the release and absorption of Ca^2+^ in the sarcoplasmic reticulum and maintains sarcoplasmic reticulum Ca^2+^ homeostasis [36]. A common variant of HRC, Ser96Ala, has been identified as an effective biomarker for triggering malignant cardiac arrhythmias [37]. Genetic deletion of interleukin-1*α* reduces ER stress-induced CHOP expression in macrophages and attenuates the progression of atherosclerosis in apoE-deficient mice [32]. TMED proteins are cargo-recruiting factors which secreted by ER stress-related proteins [38]. In addition to the aforementioned ER-related genes, we have identified the differential expression of these ER-related genes in CAD for the first time. Therefore, we further investigate whether these differentially expressed ER-related genes can serve as potential biomarkers for CAD. This lays the foundation for exploring the role of ERS and potential mechanisms in CAD. These results showed promising diagnostic performance for atherosclerosis, with RCN2 and IL-1A suggested as potential biomarkers for CAD and CRH as a potential therapeutic target.

In the GSEA analysis of this study, GO was enriched in DNA repair, cell cycle-related pathways, and muscle contraction pathways, which are strongly associated with the occurrence of CAD [39–41]. Mutations in mitochondrial DNA, including large deletions, mutations in mitochondrial DNA oxidase-related genes, and mutations in mitochondrial tRNA genes, are important causes of CAD [39]. In addition, KEGG enrichment was found in the ECM receptor interaction pathway, and the restructuring of myocardial ECM is closely related to heart failure [42]. Studies have shown that excessive deposition of extracellular matrix proteins in the myocardium can damage the myocardial structure, disrupt myocardial excitation-contraction coupling, impair contraction and relaxation function, and thus lead to the progression of heart disease to heart failure [43]. Additionally, enriched T cell receptor signaling and chemokine signaling pathways are also closely associated with the promotion of CAD development [44–46]. Immune response plays an indispensable role in the formation and development of atherosclerosis [47, 48]. T lymphocytes are the main immune cell in atherosclerotic plaques, mainly consisting of cytotoxic CD8^+^ T cells [49, 50]. Animal studies have indicated that cytotoxic CD8^+^ T cells promote the formation of necrotic cores and aggravate the progression of adverse plaques [51]. Additionally, CD8^+^ T cells can lead to apoptosis and inflammatory reactions through the secretion of cytokines such as tumor necrosis factor-alpha and IFN-gamma [52, 53]. Moreover, many chemokines participate in the complex process of atherosclerotic plaque formation by recruiting various types of leukocytes, such as monocytes, macrophages, and T lymphocytes to atherosclerotic sites [54]. Monocyte chemoattractant protein-1, known as CCL2, is mainly secreted by inflammatory cells and endothelial cells, which triggers the recruitment of monocytes to sites of inflammation [55, 56]. In addition, MCP-6, MCP-7, MCP-8, MCP-9, and MCP-10 play a major role in the entire process of atherosclerosis, from the formation of atherosclerosis to the instability of atherosclerotic plaques [57]. The GSEA results indicate that these biomarkers are related to the development of CAD and can help identify the mechanisms of biomarker involvement in CAD development. The GSEA results indicate enrichment in pathways related to DNA repair, cell cycle, muscle contraction, ECM receptor interaction, T cell receptor signaling, and chemokine signaling, indicating their significant roles in CAD development, with mutations in mitochondrial DNA also highlighted as important causes.

In atherosclerotic plaques, many circulating immune cells with chemotactic properties are involved in endothelial injury and lipid infiltration [58]. Studies have shown that the number of CD56 natural killer cells, neutrophils, activated dendritic cells, MDSCs, and Th17 cells increased in the CAD group [59]. Neutrophils are key cells that accelerate the progression of atherosclerosis; they can raise the area and instability of plaques by releasing various cytokines and adhesion factors [60] and enhance macrophage phagocytosis of lipids and MMP-9 levels [61, 62]. Dendritic cells proliferate and activate in the presence of GM-CSF produced by endothelial cells [63] and may be involved in immune signal transduction during the process of CAD. In a previous study, deficiency of NK cell led to raised serum cholesterol levels and enlarged plaque size in mice [64]. In our research, NK cells have also shown lower levels in the CAD group. RCNA2, DERL2, RNF183, and TMED2 may potentially impact the progression of CAD through NK cells. Overall, various immune cells increase in CAD, with neutrophils playing a key role in plaque progression by releasing cytokines and adhesion factors, while dendritic cells and NK cells may also influence CAD development.

As the mechanisms of occurrence and development for CAD are immune cells and inflammatory factors [65, 66], the immune composition of peripheral blood of CAD patients frequently changes throughout the course of the disease, which is associated with acute exacerbation, remission, and stability of the disease [67–69], so the significant role of immune-related factors in CAD cannot be overlooked. This study found that there are varying degrees of correlation between eight genes and chemokines, immune suppressants, and immune activators by Pearson's correlation analysis. It was explored that these differential genes possess immune characteristics associated with CAD. In addition, we validated the expression levels of the selected eight genes by RT-qPCR in the peripheral blood of CAD patients and normal individuals. The results showed that, except for RNF183 and TMED2 which did not show significant changes, the expression levels of the other six genes were significantly altered. Although the results from qPCR and bioinformatics analysis may not be identical, we considered that two different techniques may be the main reason. Moreover, the number of samples and individual differences between clinical and database samples can also contribute to the discrepancies. Therefore, to further confirm that these genes would be therapeutic targets or biomarkers for monitoring CAD, it may require more samples and additional methods to address these discrepancies.

## 5. Conclusions

In conclusion, we identified eight differential ERS genes related to CAD which can be used as biomarkers of CAD. These findings provide a new way for disease diagnosis by exploring the role of ERS in the mechanism of CAD.

In this study, it is the first time to identify CAD in the ERS-related biomarkers and immune-related analysis, but the limitations of this study are mainly the small sample size and insufficient analytical indicators. Although we selected a sample dataset related to CAD covering diverse geographic populations, which provided a comprehensive and representative analysis, we must also recognize the limitations of this geographic diversity and the potential impact of unconsidered factors on the outcomes. Thus, in future studies, it is essential to utilize larger datasets to further validate our findings. This will supply a reliable reference for future research and application. Meanwhile, it is crucial to point out that the potential biomarker function of these genes in CAD is only based on bioinformatics analysis. Clinical application of biomarkers needs to be supported by more sample data, and further comprehensive experimental researches are imperative to explore the role of these genes.

## Figures and Tables

**Table 1:** Primer sequences of biomarkers in qRT-PCR.

**Primer**	**Sequences**
DERL2 F	CCGCCGTGCAGTTGGA
DERL2 R	CCTTCTTCTAGCATTCGACAGTAA
RNF183 F	TCTACACGCTGGACCTTGGC
RNF183 R	TGCGGAAACACTCCCTCAAA
CRH F	CCCTCAGCCCTTGGATTTCT
CRH R	GCAACACGCGGAAAAAGTTG
TMED2 F	ATATGGAAACAGAAGCTCACCA
TMED2 R	GGCTCTGTGTATTCTCTCCCG
PPP1R15A F	CTGGCTGGTGGAAGCAGTAA
PPP1R15A R	TATGGGGGATTGCCAGAGGA
IL1A F	TGGTAGTAGCAACCAACGGGA
IL1A R	ACTTTGATTGAGGGCGTCATTC
RCN2 F	TAATAATCAGGGCATTGCACAAG
RCN2 R	GGAGCTGTCTGCCATAATCTGTG
HRC F	AGTCCAGTTCGGCCACTATGTT
HRC R	TTCCTCAAATGGCTTCTATCCT
GAPDH F	CGAAGGTGGAGTCAACGGATTT
GAPDH R	ATGGGTGGAATCATATTGGAAC

## Data Availability

The datasets (GSE113079 and GSE23561) analyzed in this research were obtained from the Gene Expression Omnibus (GEO) database (https://www.ncbi.nlm.nih.gov/geo/).

## Supporting Information

Supporting Information 1 Figure S1 The GO terms enriched in eight biomarkers.

Supporting Information 2 Figure S2 The KEGG pathways enriched in eight biomarkers.
