# Supplementary figures and images for: Identification of Endoplasmic Reticulum Stress-Related Biomarkers in Coronary Artery Disease
Source: Cardiovasc Ther. 2024 Jul 3;2024:4664731. doi: 10.1155/2024/4664731 (PMC11236471; doi:10.1155/2024/4664731)

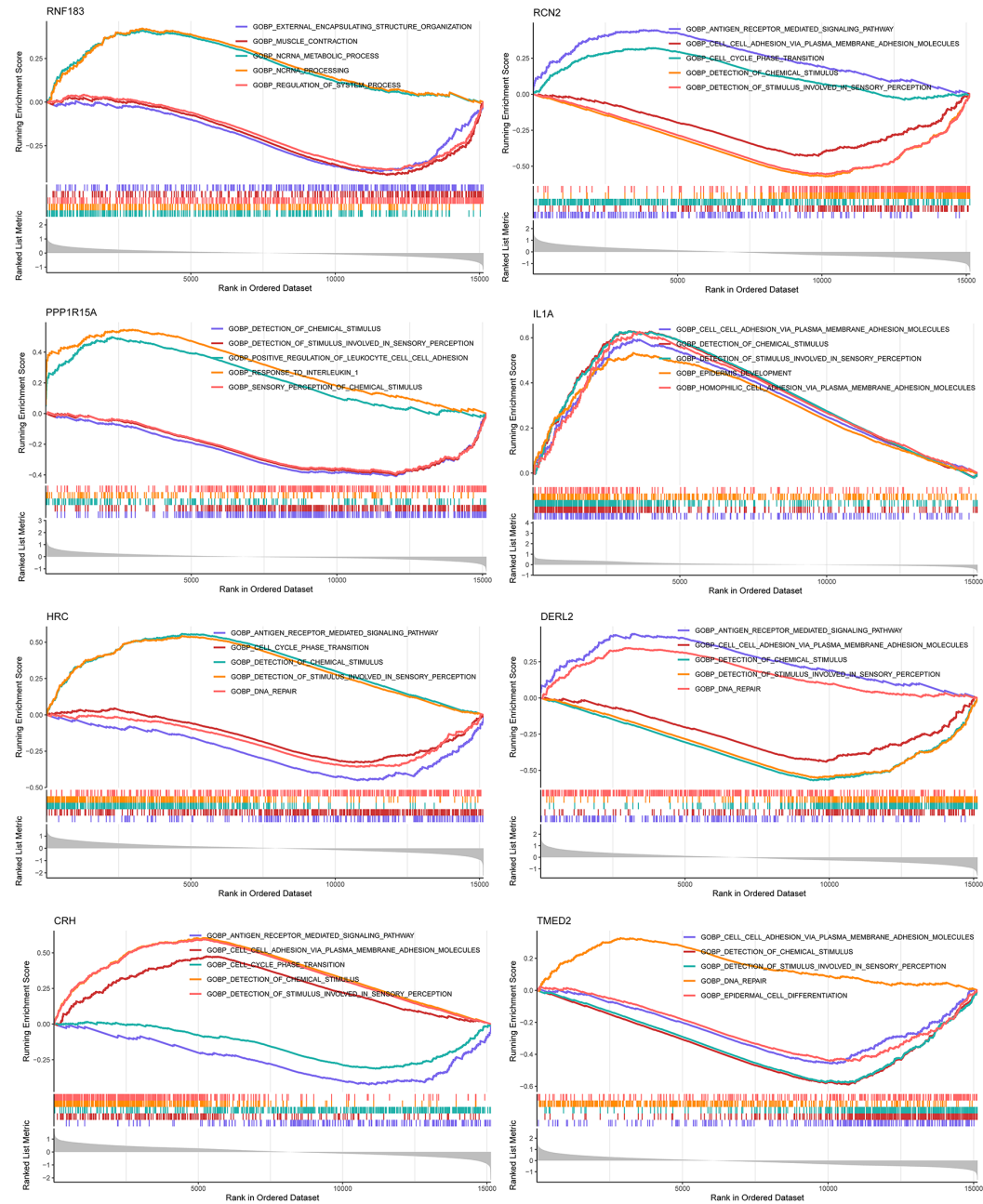

Supplement: Supporting Information 1 — Figure S1 The GO terms enriched in eight biomarkers. [file 4664731.f1.pdf]

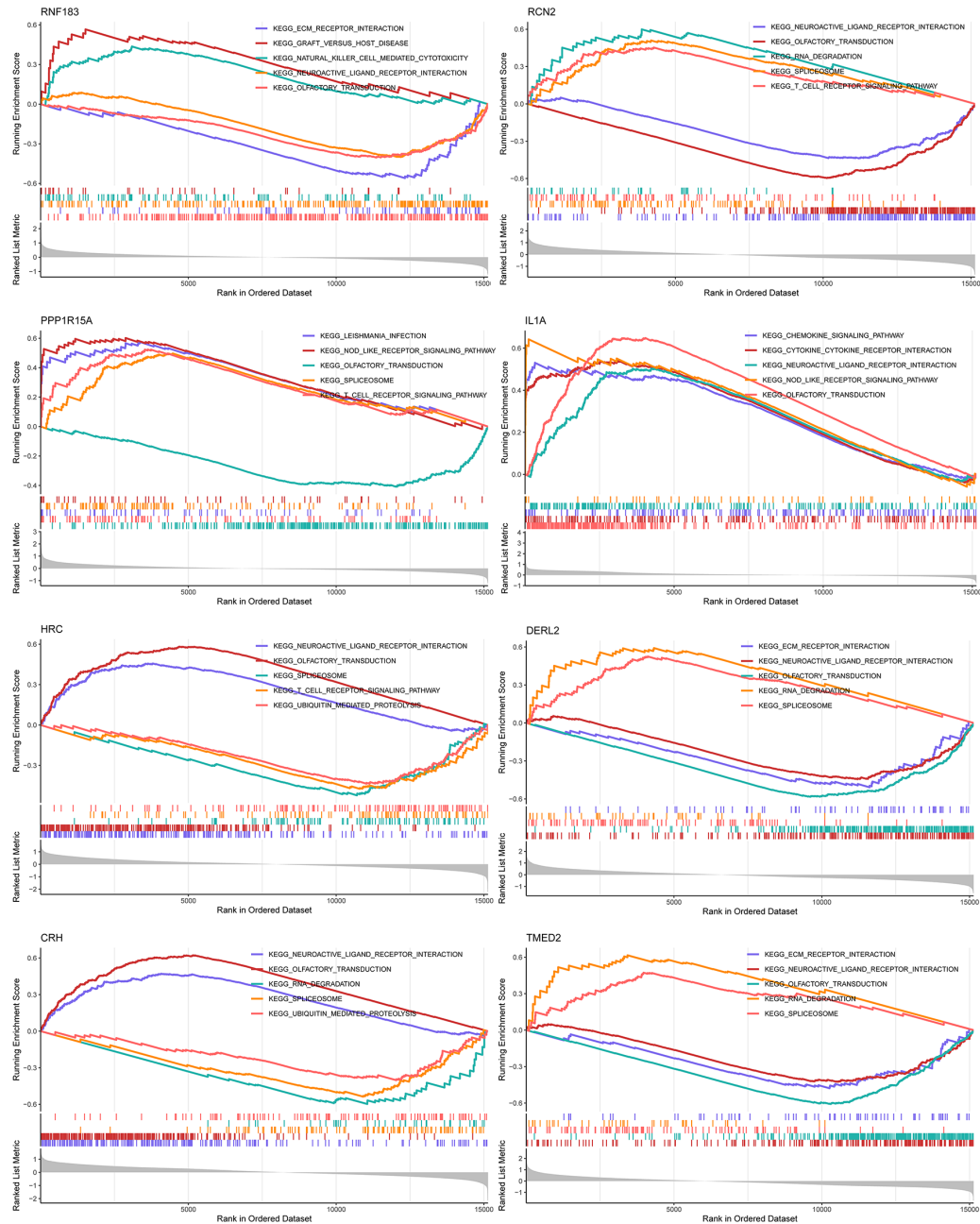

Supplement: Supporting Information 2 — Figure S2 The KEGG pathways enriched in eight biomarkers. [file 4664731.f2.pdf]
